# Using a multi-stakeholder approach to increase value for traditional agroforestry systems: the case of baobab (*Adansonia digitata* L.) in Kilifi, Kenya

**DOI:** 10.1007/s10457-020-00562-x

**Published:** 2020-11-06

**Authors:** Kathrin Meinhold, Dietrich Darr

**Affiliations:** Rhine-Waal University of Applied Sciences, Kleve, Germany

**Keywords:** Food product innovation, Community-based enterprise, Neglected and underutilised species, Multi-stakeholder approach, Rural development, Food security

## Abstract

The baobab tree (*Adansonia digitata* L.) is an integral component of many dryland farming systems in sub-Sahara Africa. Such traditional agroforestry systems can foster a variety of benefits; besides positive livelihood implications baobab can particularly address food security objectives due to its highly nutritious fruits. However, many bottlenecks persist inhibiting the broader potential of indigenous trees in farming systems and their increased utilisation and commercialisation. We suggest that traditional farming systems with baobab trees can be advanced by stimulating the emergence of local markets for baobab products while promoting businesses and innovations aimed at meeting the arising market demand. Increasing the perceived value of local agroforestry products in combination with facilitating additional commercialisation pathways will in turn lead to food security and livelihood benefits. Using a multi-stakeholder approach such considerations were put into practice in Kilifi, Kenya, by initiating a community-based enterprise development producing high-quality baobab powder and oil. Initial results demonstrate behavioural changes, an improved practical knowhow with regard to baobab management and utilisation, and an increased consumption of baobab, which may already contribute to food security objectives. Baobab is increasingly seen as a valuable resource as opposed to ‘food for the poor’ and a tree possessed by evil spirits. This may lay the groundwork for further value addition activities and enterprise development in the communities. With baobab being a common, yet so far underutilised feature of local farming systems in Kilifi, activities based on its increasing commercialisation can be complementary and easily integrable to prevailing livelihood strategies.

## Introduction

Natural or traditional agroforestry systems created by purposeful retention of indigenous trees on farmers’ land can foster a variety of benefits, including the provision of ecosystem services or positive livelihood impacts (Amare et al. 2019; Assogbadjo et al. 2012). By integrating indigenous trees in their production system rural farmers can access additional income sources, directly benefit from nutritious food products, and increase their resilience with regard to market or climatic shocks (Leakey and van Damme 2014; Reed et al. 2017). Furthermore, traditional agroforestry systems can help maintain tree and associated biodiversity (Fifanou et al. 2011), reduce soil erosion and improve soil characteristics, increasing crop yield and household food availability year-round (Apuri et al. 2018; Félix et al. 2018).

However, despite increasing awareness of such benefits, commercialisation of indigenous plants in Africa remains limited (van Wyk 2011). Indigenous fruits are often only processed on a small scale and into few products (Nitcheu Ngemakwe et al. 2017). Many bottlenecks exist limiting the potential of and benefits from indigenous fruits produced in traditional agroforestry systems (Jamnadass et al. 2011). Challenges include market insufficiencies and failures such as limited demand, inadequate supply and marketing channels or supply control mechanisms (Gruère et al. 2006; Leakey et al. 2005; Meinhold and Darr 2019). In new markets further constraints lie in low perceived returns to contributions which inhibits stakeholders to take part (Lee et al. 2018). Regulatory frameworks in sub-Saharan Africa often do not promote small enterprise development with laws being bureaucratically, weakly or randomly implemented and enforced (Rogerson 2001). Further reasons impeding successful commercialisation of underutilised plant species by rural producers include lack of financial resources and skills such as entrepreneurial capabilities (Meinhold and Darr 2019), lack of interest and acceptance in indigenous fruits (Bvenura and Sivakumar 2017), and too stringent or conflicting regulations with regard to tenure arrangements or trade (Wynberg et al. 2015).

Thus, there is a need to unlock the seemingly hidden potential of indigenous tree species used in traditional agroforestry systems in order to enhance food security, livelihoods, and increase resilience for future challenges. Learning from successful traditional agroforestry systems with neglected indigenous trees may suggest a model for sustainable development (Nair et al. 2017). We suggest that agroforestry practices involving underutilised tree species can be enhanced by stimulating the emergence of markets for products provided by these trees and the development and promotion of businesses and innovations aiming to meet the arising market demand. The foundation lies in increasing the perceived value of local agroforestry products in combination with facilitating additional commercialisation pathways for local producers and processors. This in turn will lead to food security and livelihood benefits. Against this background and using activities surrounding the baobab tree in Kilifi, Kenya, as a case study with regard to indigenous tree species in traditional farming systems, this paper aims to develop and evaluate this concept further and showcase how it can be put into practice.

## The baobab tree

Due to its particular potential for food and nutrition security as well as its relatively easy cultivation and widespread distribution, the baobab (*Adansonia digitata* L.) is an ideal candidate to study indigenous tree species in traditional farming systems. For such reasons it has also been identified as a priority species for domestication (Leakey 1999; Sanchez et al. 2010). The tree occurs naturally in the savannahs and savannah woodlands of sub-Saharan Africa, most commonly in semi-arid to arid regions (Wickens and Lowe 2008). It is often an integral component of dryland farming systems of these regions, which is also illustrated by the strong historical connection between human habitation and the distribution range of the species. Evidence suggests that baobab trees have been introduced and planted around homesteads and settlements across the African continent for centuries (Duvall 2007). Additionally, useful tree species such as the baobab are typically preserved by farmers (Teklehaimanot 2004). Consequently, baobab density has been shown to be higher in villages and fields in contrast to natural plains or rock outcrops (Dhillion and Gustad 2004; Venter and Witkowski 2010). The trees seem to be well preserved in such communal areas although land-use intensification may put this at risk (Schumann et al. 2010). Although detailed research on agroforestry systems involving the baobab is scarce, presence of baobab trees has been shown to be effective in combination with the production of taro and millet (Sanou et al. 2012).

Agroforestry systems involving baobab are of particular interest considering food security objectives. The fruit features particular nutritional properties including relatively high levels of Vitamin C and selected minerals, especially Calcium, and phytochemicals such as polyphenols (Chadare et al. 2009; Coe et al. 2013). The fruit pulp, being naturally dry when the fruit is ripe, can easily be used as an ingredient to enrich food products such as cereals, snack-bars, and cookies, thus offering the opportunity to increase nutrient intake and address micronutrient deficiencies (Gabaza et al. 2018; Mounjouenpou et al. 2018). Baobab enriched bread has been shown to reduce starch digestion and glycaemic response in humans (Coe et al. 2013). Besides being a nutritious food source, baobab offers numerous other uses including provision of medicine, fodder, handicrafts, and significance in cultural ceremonies (Gebauer et al. 2016; Kamatou et al. 2011). Furthermore, baobab fruit commercialisation can be an important income source for smallholders; with the additional income being spent on other food products, baobab can also indirectly contribute to food security objectives (Venter and Witkowski 2013). In Kenya, where baobab marketing is still in its infancy, a recent value chain study concluded that an increased commercialisation and improved value chain integration could increase income, particularly for women (Jäckering et al. 2019). To promote baobab utilisation in Kenya it has been suggested to ensure better availability, accessibility, and affordability of baobab products as well as undertake efforts with regard to product packaging, labelling and raising awareness on nutritional benefits (Kiprotich et al. 2019).

## Conceptual framework

**Fig. 1:**
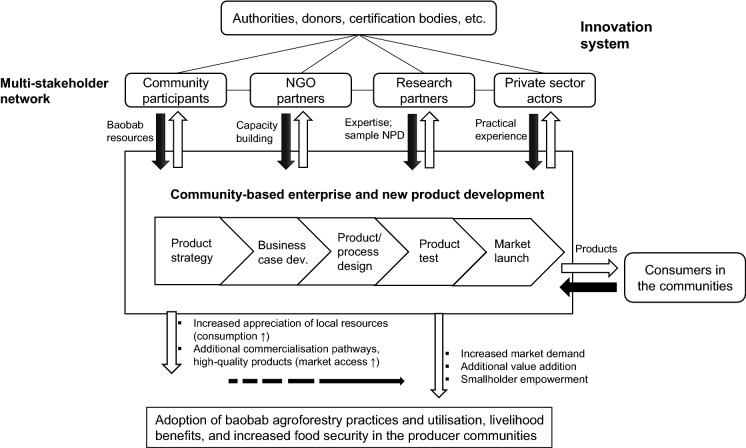
Overall conceptual framework

Scaling up agroforestry innovations is far more complex than simple transfer of information and technologies (Franzel et al. 2001). It is widely acknowledged that an enabling environment is needed to foster innovations and broader, long-term adoption of novel practices in communities (Makate 2019; Thomas et al. 2018). Conducive policies and institutional frameworks should ideally support environmentally and socially desirable practices, such as making increased use of traditional agroforestry systems. The development of demand and markets for agroforestry products and initiation of business dynamics and opportunities to serve these could be one instrument within such frameworks. Improving market access for smallholders can lead to higher income and food security (Gyau et al. 2014), whereas special attention should be drawn to often overlooked local markets (Shackleton et al. 2007). Considering market opportunities is critical to the success of agroforestry innovations; focusing solely on (technical) innovations aimed at enhancing productive output is seldom sufficient (Russell and Franzel 2004). Market emergence can also be boosted by consumers as opposed to solely private sector players (Martin and Schouten 2013). Taking these factors into consideration, and building on an overall innovation systems framework the authors suggest to further build on new product and community enterprise development principles in a multi-stakeholder approach to enhance market creation and effectiveness (Fig. 1). As such, smallholders may be in a better position to integrate technical, organisational, financial and marketing innovations.

Innovation, the development and diffusion of technologies and practices which are new in a given context (Aubert 2005), stands at the heart of the framework since it is widely regarded as key for economic growth and employment, also for rural regions (Rametsteiner and Weiss 2006). Innovation originates from complex and multidimensional dynamics and interactions encompassing a variety of actors, knowledge and skillsets, which may not only stem from within an enterprise, but also external organisations (Laperche et al. 2008). As linear approaches to innovation may overlook important elements due to the sheer complexity, there has been a rise in system’s thinking. The (agricultural) innovation systems perspective provides a holistic and comprehensive view on innovation, the actors and factors involved, possible constraints, and types of innovations offering potential solutions (Menary et al. 2019; Schut et al. 2016b). The approach emphasises the collective nature of innovation and the alignment of technical, social, institutional, and organisational dimensions in the process (Kilelu et al. 2013). In practice, innovation system thinking has driven interventions such as innovation platforms to enhance agricultural innovation. These can be considered dynamic, multi-stakeholder initiatives, where a variety of actors can come together to exchange knowledge, skills and resources, in order to take action and solve a common problem in a complex setting (Tenywa et al. 2011). Increased social capital is seen as being the prime mediator for change (Davies et al. 2018) and, when well executed, innovation platforms have been shown to be effective for scaling up agricultural innovations (Eneku et al. 2013; Sanyang et al. 2016).

While innovation theories provide a solid foundation, we suggest to add concepts from new product (NPD) and community-based enterprise (CBE) development to strengthen applicability for agroforestry products using indigenous tree species in rural areas. NPD covers processes necessary to bring a new product to the market, including developing a product strategy, developing the business case, designing the product and process, testing the product and ultimately production and market launch (Tzokas et al. 2004). Effective NPD is seen particularly important for the food industry where estimates suggest that up to 90% of products fail within one year of introduction (Rudolph 1995). Collaboration with external partners can have a positive influence on the NPD process (Mishra and Shah 2009; Mu et al. 2017). Strategies for successful NPD in the food and particularly functional foods sector include a greater integration of market and consumer knowledge and the use of cooperative networks with multiple external partners (Khan et al. 2013; Stewart-Knox and Mitchell 2003). CBEs—which can be understood as institutions through which community groups or members actively produce goods or services in response to market demands, generating income, social returns, and other assets to benefit the communities (Macqueen 2008; Molnar et al. 2008)—may be a promising pathway to translate outcomes from NPD and innovation to livelihood benefits. CBEs can take various forms with different business models (Ambrose-Oji et al. 2015). Practices seen as beneficial for CBE effectiveness include targeted employment of marginalised groups and representation in governance, or interaction with a large array of supporting agents (Macqueen et al. 2020; Torri 2010). A diverse partner network can help CBEs in fundraising, technical training and support in business networking and marketing, or innovation and knowledge transfer (Seixas and Berkes 2010).

## Study area and methods

**Fig. 2:**
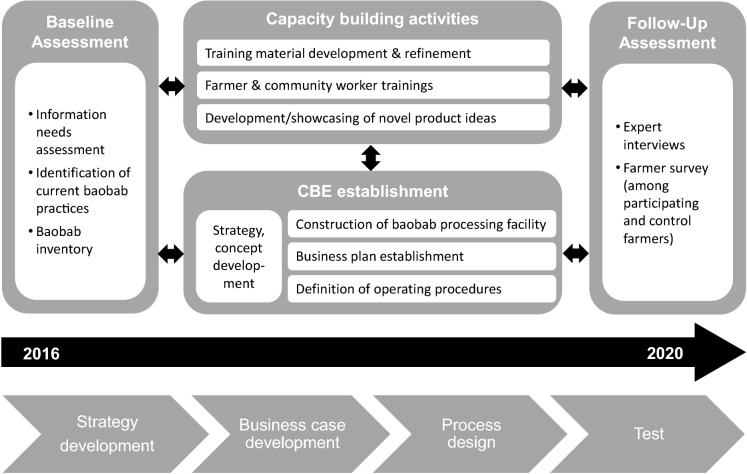
Overview of activities undertaken in Kilifi, Kenya

Activities to put above framework into practice and enhance baobab utilisation and commercialisation in Kilifi, Kenya commenced in autumn 2016. Comprising actors such as research institutions active in food technology, agroecology, nutrition, and agricultural economics, NGO partners, and private sector parties from the baobab industry a dynamic initiative was formed. The current study covers the activities until early 2020, whereas a pilot processing facility was fully established and an initial assessment conducted. Figure 2 illustrates the main steps undertaken to stimulate demand and increase market opportunities for baobab, as explained in the following subsections.

### Study area

All activities were conducted in Kilifi County in coastal Kenya, due to the high prevalence yet little utilisation of baobab and high levels of food insecurity in the region. Annual mean temperature (average annual precipitation) ranges from 21 to 30 °C (900 mm to 1300 mm) at the coast and 30 °C and 34 °C (300 mm to 900 mm) in the hinterland (County Government of Kilifi 2018). Rainfalls occur in a bimodal pattern with the short rains occurring between October and December and the long rains between March and May. Baobab trees are common in the County. Compared to inland regions, trees from Kilifi have been shown to be more high-yielding and produce larger, more sour tasting fruit (Omondi et al. 2019). Agriculture, tourism and fishing can be considered main economic activities. A large proportion of the predominantly rural population engages in subsistence family farming, with dominant crops including maize, cassava, and mung beans; main horticultural crops include cashew nut, coconut, and mango (County Government of Kilifi 2018). Poverty and food insecurity are highly prevalent. For example, poor dietary diversity scores and, amongst children, high rates of stunting and wasting have been observed (Momanyi et al. 2019).

### Baseline assessment

A baseline household survey to assess community capacity was conducted in the Majajani/Mavueni sub-location, the core implementation area, with a total population of 8005. Using a systematic random sampling technique 120 households (10.3%) were selected and primary data collected using a semi-structured, previously tested questionnaire. In addition, key informant interviews were conducted targeting people already engaged with baobab processing as well as NGOs and county agencies active in natural resource utilisation, identified via snow-ball sampling. Descriptive statistics were applied to analyse quantitative data, whereas qualitative data was subjected to content analysis to reveal further information on perception and knowledge levels about baobab and current utilisation and management practices.

### Capacity building initiative

The baseline assessment enabled identification of knowledge gaps concerning baobab and the development of a tailor-made capacity building strategy, including the establishment of customised training manuals for farmers and community extension agents. Involved scientific and private partners provided input to ensure information on nutrition, food technology, and marketing was not only scientifically sound but also reflecting practical realities. In total 60 community extension agents and 60 farmers were trained between December 2017 to September 2019. Participating farmers were purposefully selected through village leaders and networks of extension agents based on whether they possessed baobab trees and their willingness to become involved in baobab processing activities. Farmer trainings covered aspects such as baobab nutritional value, post-harvest management, product preparation and marketing. Four additional community members were also trained in operating the central processing facility. Trainings for community extension agents intended to not only strengthen their capacity, but also enable them to distribute knowledge further into the communities to ensure that, once demand of baobab increases, further farmers can be linked to the CBE operation. Alongside these activities novel baobab products, developed by food and process technologists, were showcased and sensory evaluations conducted. This intended to a) demonstrate the versatile use of baobab in both sweet and savoury food preparations to stimulate home consumption and subsequently demand and b) give ideas for future product diversification and small-scale business opportunities based on baobab.

### CBE development

Simultaneously with the capacity building initiative the actual establishment of the CBE was initiated. Results from the baseline assessment coupled with the expertise of the different actors involved informed the overall strategic outline of the CBE, taking into account the local conditions. The BAOFOOD research project consortium formed the basic partner network, however, over time links to other initiatives working on high-quality baobab processing in neighbouring countries (e.g. Mozambique, Zimbabwe, or South Africa) were established for further advice. Private sector players were particularly important to establish a detailed business plan and standard operation procedures (SOPs), whereas research partners provided guidance on aspects such as baobab resource base, value chain setup or product development and nutritional implications. CBE development activities continuously informed and were closely linked to the capacity building activities, e.g. by focussing on product handling skills and food hygiene practices to be fulfilled during operation. All farmers having participated in the trainings were registered as contact farmers enabling them to supply pre-processed baobab to the central processing facility in Kilifi, where production of baobab powder and oil takes place. Pre-processing entails the selection and correct storage of high-quality fruit, as well as extracting the pulp-on-seed after a defined timeframe in the communities. Construction activities of the central processing facility, including both the actual premises as well as the commissioning of processing machinery such as oil mill and powder extractor were concluded in September 2019, enabling the CBE to be operational for the subsequent baobab harvesting season. However, additional activities are needed in future particularly regarding empowerment of community members and generation of a self-sufficient operation.

### Follow-up assessment

To assess effects to date of the CBE development and the capacity building program, a further survey was conducted in April/May 2020. All community members who were involved in the intervention were interviewed, including both the 60 baobab suppliers as well as the 4 processors at the CBE. Furthermore, 59 farmers from the same region acted as a control group, identified via a simple random sampling approach. Semi-structured interviews were used to gain insights on changes in baobab utilisation and management as well as potential benefits community members may have already gained. Descriptive and comparative analyses were conducted using the Statistical Package for the Social Sciences program (SPSS) to gain a better understanding of changes which have occurred during the intervention. Data from the survey was complemented by expert interviews of key stakeholders involved in the CBE.

## Results

### Baseline assessment

The baseline assessment demonstrated low education levels (42.5% of household heads illiterate, further 43.4% primary school level), main livelihood activity was crop farming and temporary food shortages a regular problem (70% of respondents). The majority (69%) owned naturally occurring baobab trees on their land. Approx. 1/3 of respondents had been involved in making a baobab product, yet only a minority was engaged in any tree management activities other than harvesting. Farmers more likely to harvest baobab included ones with larger farms, of younger age, and with more knowledge concerning baobab products and management practices. Knowledge levels were generally low and closely correlated to prevailing practices. On a commercial level, one processed food product (“*mabuyu*”) was identified, consisting of the dry baobab pulp and seed prepared in a mix of sugar and food colouring, targeting mainly children as consumers and sold in village kiosks or by the road side. Besides these and a use in juice or porridge, known to 93.3%, 40.0%, and 39.2% of respondents, respectively, awareness for other baobab products and their nutritional value was very low. In regional markets a few non-food baobab products were identified, including drums and lampshades made from shells, paper from bark, or soap from oil, targeting mainly tourists or middle to high income earners. No organised community group was found dealing with baobab either for value addition or selling raw material.

Overall, the results pointed to a basic, yet expandable baobab utilisation. Reasons cited for a not more widespread use and management included negative perceptions associated with the baobab tree in coastal Kenya (hosting of evil spirits), or the lack of information concerning utilisation practises and benefits. Nevertheless, 68.8% of respondents expressed interest to engage in more intensive and/or improved management and utilisation of baobab in the future. Information needs identified were particularly high with regard to storage, processing both for sale and home consumption, marketing, as well as nutritional and health benefits. Temporary food shortage was significantly linked to higher information needs regarding processing for sale (*p* < 0.05). Thus, sensitising farmers about the commercial potential of the tree, the variety of uses, and its nutritional benefits may promote its utilisation, change attitudes and beliefs about the tree, and increase household uptake of baobab products.

### Established overall strategy of the CBE

Since small-scale baobab commercialisation already existed a focus was put on producing bulk, high-quality raw materials (baobab fruit powder and oil). To ensure hygienic conditions the central processing facility consists of several closed structures for storage, processing as well as changing rooms for staff. By partnering with existing regional microenterprises further local value addition should be generated and innovation and business development stimulated. This may be aided by the strategic location of the central facility in a tourist hot-spot and supporting activities by the involved NGO, in which hands the CBE currently lies. The target was set to initially focus on local and regional markets as well as gradual growth—allowing to perfect the production model, supply chain and quality assurance along the way and ensuring a stable base for production easily expandable with growing demand, including the integration of more farmers. Experience from other countries showed that early concentration on export had a high risk of creating a boom-bust situation and opportunities from local markets are—despite the opportunities arising from a growing middle class (Darr et al. 2020)—often overlooked.

The management procedures of the CBE entail that after local, community-based harvest and primary processing at rural collection centres, final processing into baobab powder and oil takes place at the central facility, all conducted by trained community members. All steps in the production process, from fruit collection to cleaning processing equipment are regulated via SOPs and were topics during capacity building. Only farmers having undergone registration and training are able to supply baobab and receive annual supply contracts, to be renewed if harvesting, field processing and storage procedures were adhered to. Farmers are to be paid on a cash on delivery basis upon satisfactory quality and weight control. With an anticipated purchase price of 0.23 USD/kg of pulp, it is estimated that the average farmer could be earning an additional USD 84 per season. Pulp is to be purchased from September to December with powder processing to take place between January and March during the dry season. Initial scale of production is 5 t of baobab powder and 500 kg of oil per year, equalling 25 t of pulp-on-seed, to increase to 10 t of powder after 7 years (~ 15% annual increase). As demand increases, the CBE will expand the radius of the targeted area or replicate baobab processing activities in other Kenyan regions.

With regard to governance, a clear shareholding structure is envisaged, whereas community members will hold part of the shares and profits will be distributed in line with shareholdings. The board of directors, comprising partnering shareholders, will oversee the realisation of the business plan in a democratic fashion and maintain an active advisory oversight role to offer expertise in areas such as factory operations, marketing and supply chain development, commercial operations and certification. At present, however, the CBE can dominantly be regarded a research-private sector initiative. Community members have, nevertheless, expressed interest for further involvement and already committed land and materials for the construction of local storage shelters. Further participation was inhibited by lack of business experience as well as the financial risk this would entail. As of now, the CBE has survived from donor funding, in total approx. 100,000 USD, of which 28% were construction, 5% equipment, 44% training, and 23% consultancy costs. To date, the operation is not yet economically self-sustainable, although it is anticipated that after the first year of operation profits will be generated. Direct operating costs have been kept low by maintaining relatively short supply chain and logistics, whereas an emphasis has been put on marketing costs as this has been considered particularly important since dealing with novel products.

### Initial outcomes

Table 1 presents basic demographic data of the farmers involved in capacity building and linked to the CBE as well as control farmers. Whereas trained and control farmers ranked similarly in household size, gender, and income sources, significant differences (*p* < 0.05) were observed in terms of baobab tree number on farms, age and education. This may be explained by the purposive selection of farmers.

**Table 1:** Demographics of trained and control farmers

	Trained farmers	Control
Age [years]	48.93 ± 14.25	40.98 ± 15.65*
Gender [% female]	53.3	57.6
Household size	8.72 ± 3.99	7.98 ± 3.50
Education [% no or only primary education]	88.3	78.0*
Farm size [acres]	10.64 ± 18.45	6.25 ± 11.85
Baobab trees on farm	10.20 ± 17.66	5.29 ± 6.30*
Income source [% crop farming ranked most important]	71.7	61.0

*Significant results, *p* < 0.05

Differences with regard to baobab between the two groups are illustrated in Table 2. Almost all trained farmers reported to have changed at least some of their practices concerning baobab management and utilisation; however, also the untrained farmers changed practices, although to a lesser degree. Over 90% of trained farmers reported to have changed the way they select, store and process baobab fruit, focussing on ripe, brown, undamaged fruits and maintaining hygienic practices; amongst the control 49.2% reported changes in fruit selection, 30.5% changed their storage practices by building structures, and 28.8% reported changes in primary processing of fruit. Both trained and control farmers reported to consume baobab more often at home (63.3% and 37.3%, respectively), e.g. as juice or adding it to soups. The decision for increased consumption was mainly driven by perceived nutritional benefits. Approx. half of both groups reported to have started experimenting with baobab, e.g. by making new products or using it as a food additive (48.3% and 50.8%, respectively). Local farmer cooperatives have started to integrate baobab in locally produced yoghurt or cookies, although no novel products have appeared in local markets yet. 43.3% of trained farmers indicated that the market price for baobab has recently changed, 84.6% of which reported a price increase (control: 25.9% and 93.3%, respectively). Although 68.3% of trained farmers (control 44.1%) had already been involved in selling baobab, predominantly to local traders, 30% (13.6%) of farmers reported using baobab more for sales and a total of 13 farmers (21.7%) started selling baobab only after the training. This is despite the fact that actual purchases from the CBE had not taken place yet. Nevertheless, farmers rated potential benefits from the CBE highly, their main motivation to participate was primarily to gain knowledge on baobab, followed by market, income, and labour opportunities. To increase the success rate, farmers suggested further capacity building efforts, inclusion of more farmers, as well as regular communication efforts.

**Table 2:** Comparison between trained and control farmers in the study region

Factor	Trained farmers		Control
*Knowledge level*^*a*^			
Baobab trees general	4.33 ± 0.63 (1.70 ± 0.79* before training)		1.61 ± 0.77
Harvesting methods	4.22 ± 0.78 (1.53 ± 0.75*)		1.71 ± 0.81
Fruit selection	4.40 ± 0.76 (1.82 ± 0.83*)		2.15 ± 0.76*
Storage practices	4.38 ± 0.64 (1.40 ± 0.72*)		1.78 ± 0.91*
Baobab processing	4.37 ± 0.80 (1.37 ± 0.64*)		1.80 ± 0.83*
Hygienic practices	4.28 ± 0.74 (1.27 ± 0.48*)		1.66 ± 0.90*
Nutrient content	4.12 ± 0.83 (1.22 ± 0.41*)		1.27 ± 0.66
Product variety	4.42 ± 0.72 (1.80 ± 0.73*)		2.05 ± 0.92
Economic benefits	3.88 ± 0.90 (1.42 ± 0.59*)		1.76 ± 0.70*
*Baobab consumption*			
Increased family use	63.3%		37.3%*
Current consumption levels			
Never	30.5%		60.4%*
1–2 times per week	33.9%		11.3%*
3–5 times per week	28.8%		22.6%
Daily consumption	6.8%		5.7%
*Baobab sales*			
Percentage selling baobab	68.3%		44.1%*
Recent increased use for sale	30.0%		13.6%*
Average weekly baobab income *(All farmers/farmers selling baobab)*			
Less than 100 KES	18.3%/28.9%		3.4%*/8.0%*
100–300 KES	23.3%/36.8%		16.9%/40.0%
300–600 KES	8.3%/13.2%		6.8%/16.0%
600–900 KES	1.7%/2.6%		6.8%/16.0%
More than 900 KES	11.7%/18.4%		8.5%/20%
Income contribution *(All farmers/farmers selling baobab)*			
Less than 25%	50.0%/88.2%		40.7%/96.0%
25–50%	6.7%/11.8%		1.7%/4.0%
*Baobab supply*			
Baobab collection per season *(All farmers/farmers collecting)*			
	*Before CBE*	*After CBE*	
1–100 kg	23.3%/31.1%	(11.7%/28.0%)	30.5%/46.2%
100–300 kg	20.0%/26.7%	(13.3%/32.0%)	20.3%/30.8%
300–600 kg	18.3%/24.4%	(13.3%/32.0%)	6.8%/10.3%
600–900 kg	8.3%/11.1%	(3.3%/8.0%)	6.8%/10.3%
More than 900 kg	5.0%/6.7%	(0%/0%)	1.7%/2.6%
Days spent			
1–7 days	46.7%/62.2%	(20.0%/50.0%)	52.5%/86.1%*^b^
7–14 days	13.3%/17.8%	(3.3%/8.3%)	6.8%/11.1%
14–28 days	8.3%/11.1%	(5.0%/12.5%)	0%/0%*^c^
More than 28 days	0%/0%	(11.7%/29.2%*)	1.7%/2.8%*^d^

^a^Rating scale 1–5; 1 equalling little knowledge, 5 vast knowledge, ^b^significant both for values compared to before and after CBE establishment; ^c^significant only for values compared to before CBE establishment; ^d^significant only for values compared to after CBE establishment

*Significant results, *p* < 0.05

## Discussion

Although the long-term effectiveness of the undertaking remains to be seen and the CBE is still a work in progress, the initial results are encouraging. Baobab subsistence use has increased locally, which due to its nutritional profile can directly contribute to food security objectives. Behavioural changes and an improved practical knowhow indicate that the overall perception is changing towards baobab being a valuable resource as opposed to ‘food for the poor’ and a tree possessed by evil spirits. Thus, the first step of the conceptual framework developed has been reached, laying the groundwork for an increased future demand for baobab products, and, ultimately, for the success of the CBE. Detailed effects on income, employment and market creation are, however, yet uncertain with the CBE not fully having started operation. Experiences from other countries with an already higher degree of baobab commercialisation show that annual mean income from baobab pulp and seed sales was in the realm of USD136 ± 14 (Venter and Witkowski 2013), while our estimates are in the range of USD84. Such supplemental income, however, can be a vital contribution to livelihoods of marginalised communities. With the current price for baobab pulp and seed in Kenya being in the realm of 0.07 USD/kg (Jäckering et al. 2019) in contrast to the anticipated sales price of 0.23 USD/kg with the CBE this will be an attractive opportunity for smallholders.

An increasing baobab commercialisation, however, may also lead to overharvesting of the resource or threaten subsistence use and livelihoods (Buchmann et al. 2010). These risks are currently perceived as low for the study region. Baobab populations in Kenya have been shown to be healthy, stable, and rejuvenating (Fischer et al. 2020). In comparison to Western Africa, where baobab is more highly utilised, baobab in Kenya can yet become a more important part of local diets. To nevertheless not undermine subsistence use when demand increases, the groundwork has been laid to enable benefit-flows to small-scale producers by set management and governance principles. These were strongly aided by the experience from PhytoTrade Africa, a natural product trade association with broad experience in connecting smallholders to natural products markets and enabling market opportunities while achieving livelihoods benefits (Welford and Le Breton 2008). Set standards and procedures will also ease future certification, which, alongside capacity building efforts, has been put forward as a potential solution for sustainable and ethical trade of baobab (Buchmann et al. 2010).

The comparison with other CBE initiatives in natural resource management, although complicated by the variety of business models, demonstrated that challenges are often similar. Issues such as financial challenges, including the dependence on external investments or lack of management or marketing skills on the ground are common problems (Ambrose-Oji et al. 2015; Pandit et al. 2009). Strategies put forward to overcome such challenges include the development of entrepreneurial and organisational skills in farmers, a focus on strategic partnerships and networks, special attention to organisational set-up and governance mechanisms to ensure equal benefit distribution and conflict resolution, and following a holistic approach considering the local setting (Macqueen et al. 2020; Steiner and Teasdale 2019; Torri 2010). Many such aspects could be addressed in our case study due to the foundation in innovation system thinking. Special attention has been put on collaboration and keeping an integrated approach, whereas the inclusion of both experienced private sector players from the baobab sector and research partners has proven especially helpful, e.g. in defining organisational setup, management procedures, and capacity building efforts. Other aspects need further attention in future to ensure the CBE’s long-term success, in particular inclusion of socially marginalised groups in decision-making procedures and leadership, and continuous skill development in the communities, particularly with regard to entrepreneurship and business development—issues to be tackled in the next phase of the intervention.

Illustrated approach also shares many similarities with innovation platforms, as these should provide opportunities for local innovations while nourishing introduced ones (Tenywa et al. 2011) or the emphasis on effective, strategic partnerships. Although effectiveness of innovation platforms can strongly differ, they have been shown to foster innovation and market creation. For example, Pamuk et al. 2014 demonstrated that they aided crop management innovation adoption and, according to Mumbeya et al. 2020, participation in innovation platforms led to an increase of rural female farmer income via improved market access. Innovation platforms have also led to increased farmer’s technical knowhow, enhanced farm productivity, and behavioural changes (Davies et al. 2018; Sanyang et al. 2016). Nevertheless, underlying goals of innovation platforms often go far beyond such outcomes, aiming to change prevailing institutional regimes to create opportunities for smallholders (Hounkonnou et al. 2018). By creating opportunities for the different actors in an innovation system to connect, co-evolution should be fostered (Kilelu et al. 2013). This entails—originating from traditional, technology-centred, linear approaches—a paradigm shift in agricultural research for development, requiring broader structural changes far beyond individual projects (Schut et al. 2016a). Whether this is achieved in practice is disputed, yet such considerations generally go beyond our approach. Due to the inclusion of CBE and NPD elements, the latter of which has been shown to help rural groups generate diversified, high quality products (Hernández Girón et al. 2004), it is more streamlined. Detailed financial projections in our approach during business plan establishment based on expected revenues and costs for ten years after initial completion of the CBE may foster its economic sustainability. A step by step approach with special consideration on overhead costs and profits generated is important to reduce dependence on external funding in the long run. Nevertheless, there is yet a need to assess the respective costs and benefits of different approaches in order to enhance effectiveness of interventions (Franzel et al. 2001).

Overall, the following lessons learnt can be derived from the case study and the underlying approach. It was possible to enhance appreciation of the local farming population with regard to baobab, inducing more intense consumption and change in management practices. The developed CBE strategy benefited strongly from experiences of baobab-processing initiatives from other countries as well as academic and non-academic collaborators engaged in the initiative, providing scientific, local, and business knowledge and experience. By careful management of expectations of farmers it was possible to sustain their engagement despite the long timeline, which was necessary to thoroughly develop underlying strategies and management principles. Although arising benefits will be supplemental, these nevertheless can be an important contribution as marginalised groups, women were targeted. Baobab is an ideal candidate for product development activities due to its versatile use as an ingredient for both sweet and savoury foods while holding a nutritional profile which can contribute to food security. The approach may be more cost-efficient and sustainable than other initiatives targeting innovation, although more research is required in this regard. However, it has to be acknowledged that without external funding the implementation would not have been feasible. Due to prevailing market failures such as environmental externalities or information asymmetries, particularly since the baobab sector in Kenya is still in early stages, further investments into its market and value chain development are necessary. The CBE alone is not in a position to take these investments and private-sector contributions may not be easy to find due to potential risks—however, the associated social benefits justify further public investments.

## Conclusion

With the complexity involved in agricultural research for development being increasingly recognised, there has been a shift from linear to more complex, integrated approaches to foster innovation in resource-poor environments. The approach developed in this piece of work, integrating CBE and NPD principles in a framework based on innovation systems and multi-stakeholder collaboration, fits right in here. It features many characteristics associated with successful commercialisation of underutilised plant species, in particular capacity building of communities, strategic partnerships with a variety of stakeholders, using simple, scalable technologies, and taking into account the local context. The practical implementation in Kilifi demonstrated, that it was possible to increase local demand and the value seen in baobab, laying the groundwork for further value addition and enterprise development in the communities. This may also contribute to maintaining or even increasing baobab agroforestry and conservation of baobab trees on farms. With baobab being a highly nutritious food source the increased consumption may already contribute to food security. Effects on income, livelihoods, or empowerment of communities, however, will probably only be seen further down the line. Nevertheless, with baobab being a common, yet so far underutilised feature of local farming systems in Kilifi, the approach offers the possibility to be complementary and easily integrable to prevailing livelihood strategies. Although projections show that additional income would only be supplemental, this can help marginalised communities diversify livelihood strategies—focussing solely on baobab as an income source would be too much a risk. The strategy on developing high quality raw material baobab powder and oil via the CBE processing operation enables additional local marketing pathways for baobab products—considering both the input as well as output resources. Thus, CBEs and the developed approach can be a promising model for the sustainable local rural development of poor populations and—being more streamlined—possibly more cost-effective than comparable initiatives, although this should be more thoroughly addressed in future research.

## References

[CR1] Amare D, Wondie M, Mekuria W, Darr D (2019) Agroforestry of smallholder farmers in Ethiopia: practices and benefits. Small Scale For 18:39–56. 10.1007/s11842-018-9405-6

[CR2] Ambrose-Oji B, Lawrence A, Stewart A (2015) Community based forest enterprises in Britain: two organising typologies. For Policy Econ 58:65–74. 10.1016/j.forpol.2014.11.005

[CR3] Apuri I, Peprah K, Achana GTW (2018) Climate change adaptation through agroforestry: the case of Kassena Nankana West District, Ghana. Environ Dev 28:32–41. 10.1016/j.envdev.2018.09.002

[CR4] Assogbadjo AE, Glèlè Kakaï R, Vodouhê FG, Djagoun CAMS, Codjia JTC, Sinsin B (2012) Biodiversity and socioeconomic factors supporting farmers’ choice of wild edible trees in the agroforestry systems of Benin (West Africa). For Policy Econ 14:41–49. 10.1016/j.forpol.2011.07.013

[CR5] Aubert J-E (2005) Promoting innovation in developing countries: a conceptual framework: world bank policy research working paper 3554. The World Bank

[CR6] Buchmann C, Prehsler S, Hartl A, Vogl CR (2010) The importance of baobab (*Adansonia digitata* L.) in rural West African subsistence–suggestion of a cautionary approach to international market export of baobab fruits. Ecol Food Nutr 49:145–172. 10.1080/03670241003766014 21883078 10.1080/03670241003766014

[CR7] Bvenura C, Sivakumar D (2017) The role of wild fruits and vegetables in delivering a balanced and healthy diet. Food Nutr Secur 99:15–30. 10.1016/j.foodres.2017.06.046 10.1016/j.foodres.2017.06.046 28784471

[CR8] Chadare FJ, Linnemann AR, Hounhouigan JD, Nout MJR, Van Boekel MAJS (2009) Baobab food products: a review on their composition and nutritional value. Crit Rev Food Sci Nutr 49:254–274. 10.1080/10408390701856330 19093269 10.1080/10408390701856330

[CR9] Coe SA, Clegg M, Armengol M, Ryan L (2013) The polyphenol-rich baobab fruit (*Adansonia digitata* L.) reduces starch digestion and glycemic response in humans. Nutr Res 33:888–896. 10.1016/j.nutres.2013.08.002 24176228 10.1016/j.nutres.2013.08.002

[CR10] County Government of Kilifi (2018) County integrated development plan 2018–2022

[CR11] Darr D, Chopi-Msadala C, Namakhwa CD, Meinhold K, Munthali C (2020) Processed baobab (*Adansonia digitata* L.) food products in malawi: from poor men’s to premium-priced specialty food? Forests 11:698

[CR12] Davies J, Maru Y, Hall A, Abdourhamane IK, Adegbidi A, Carberry P, Dorai K, Ennin SA, Etwire PM, McMillan L, Njoya A, Ouedraogo S, Traoré A, Traoré-Gué NJ, Watson I (2018) Understanding innovation platform effectiveness through experiences from West and Central Africa. Agric Syst 165:321–334. 10.1016/j.agsy.2016.12.014

[CR13] Dhillion SS, Gustad G (2004) Local management practices influence the viability of the baobab (*Adansonia digitata* Linn.) in different land use types, Cinzana, Mali. Agr Ecosyst Environ 101:85–103. 10.1016/S0167-8809(03)00170-1

[CR14] Duvall CS (2007) Human settlement and baobab distribution in south-western Mali. J Biogeogr 34:1947–1961

[CR15] Eneku GA, Wagoire WW, Nakanwagi J, Tukahirwa JMB (2013) Innovation platforms: a tool for scaling up sustainable land management innovations in the highlands of eastern Uganda. Afr Crop Sci J 21:751–760

[CR16] Félix GF, Diedhiou I, Le Garff M, Timmermann C, Clermont-Dauphin C, Cournac L, Groot JCJ, Tittonell P (2018) Use and management of biodiversity by smallholder farmers in semi-arid West Africa. Global Food Security 18:76–85. 10.1016/j.gfs.2018.08.005

[CR17] Fifanou VG, Ousmane C, Gauthier B, Brice S (2011) Traditional agroforestry systems and biodiversity conservation in Benin (West Africa). Agrofor Syst 82:1–13. 10.1007/s10457-011-9377-4

[CR18] Fischer S, Jäckering L, Kehlenbeck K (2020) The Baobab (*Adansonia digitata* L.) in Southern Kenya–A Study on Status, Distribution, Use and Importance in Taita-Taveta County. Environ Manag. 10.1007/s00267-020-01311-7 10.1007/s00267-020-01311-7 PMC8172389 32533325

[CR19] Franzel S, Cooper P, Denning GL (2001) Scaling up the benefits of agroforestry research: lessons learned and research challenges. Dev Pract 11:524–534. 10.1080/09614520120066792

[CR20] Gabaza M, Shumoy H, Muchuweti M, Vandamme P, Raes K (2018) Baobab fruit pulp and mopane worm as potential functional ingredients to improve the iron and zinc content and bioaccessibility of fermented cereals. Innov Food Sci Emerg Technol 47:390–398. 10.1016/j.ifset.2018.04.005

[CR21] Gebauer J, Adam YO, Sanchez AC, Darr D, Eltahir MES, Fadl KEM, Fernsebner G, Frei M, Habte T-Y, Hammer K, Hunsche M, Johnson H, Kordofani M, Krawinkel M, Kugler F, Luedeling E, Mahmoud TE, Maina A, Mithöfer D, Munthali CRY, Noga G, North R, Owino WO, Prinz K, Rimberia FK, Saied A, Schüring M, Sennhenn A, Späth MA, Taha MEN, Triebel A, Wichern F, Wiehle M, Wrage-Mönnig N, Kehlenbeck K (2016) Africa’s wooden elephant: the baobab tree (*Adansonia digitata* L.) in Sudan and Kenya: a review. Genet Resour Crop Evol 63:377–399. 10.1007/s10722-015-0360-1

[CR22] Gruère GP, Giuliani A, Smale M (2006) Marketing underutilized plant species for the benefit of the poor: a conceptual framework: EPT discussion paper 154

[CR23] Gyau A, Franzel S, Chiatoh M, Nimino G, Owusu K (2014) Collective action to improve market access for smallholder producers of agroforestry products: key lessons learned with insights from Cameroon’s experience. Curr Opin Environ Sustain 6:68–72. 10.1016/j.cosust.2013.10.017

[CR24] Hernández Girón JP, Domínguez Hernández ML, Jiménez Castañeda JC (2004) Participatory methodologies and the product development process: the experience of Mixtec craftswomen in Mexico. Dev Pract 14:396–406. 10.1080/0961452042000191213a

[CR25] Hounkonnou D, Brouwers J, van Huis A, Jiggins J, Kossou D, Röling N, Sakyi-Dawson O, Traoré M (2018) Triggering regime change: a comparative analysis of the performance of innovation platforms that attempted to change the institutional context for nine agricultural domains in West Africa. Agric Syst 165:296–309. 10.1016/j.agsy.2016.08.009

[CR26] Jäckering L, Fischer S, Kehlenbeck K (2019) A value chain analysis of baobab (*Adansonia digitata* L.) products in Eastern and Coastal Kenya. J Agric Rural Dev Trop Subtrop (JARTS) 120:91–104

[CR27] Jamnadass RH, Dawson IK, Franzel S, Leakey RRB, Mithöfer D, Akinnifesi FK, Tchoundjeu Z (2011) Improving livelihoods and nutrition in sub-Saharan Africa through the promotion of indigenous and exotic fruit production in smallholders’ agroforestry systems: a review. Int For Rev 13:338–354. 10.1505/146554811798293836

[CR28] Kamatou GPP, Vermaak I, Viljoen AM (2011) An updated review of *Adansonia digitata*: a commercially important African tree. Econ Bot 77:908–919. 10.1016/j.sajb.2011.08.010

[CR29] Khan RS, Grigor J, Winger R, Win A (2013) Functional food product development—opportunities and challenges for food manufacturers. Trends Food Sci Technol 30:27–37. 10.1016/j.tifs.2012.11.004

[CR30] Kilelu CW, Klerkx L, Leeuwis C (2013) Unravelling the role of innovation platforms in supporting co-evolution of innovation: contributions and tensions in a smallholder dairy development programme. Agric Syst 118:65–77. 10.1016/j.agsy.2013.03.003

[CR31] Kiprotich C, Kavoi MM, Mithöfer D, Yildiz F (2019) Determinants of intensity of utilization of Baobab products in Kenya. Cogent Food Agric 5:1704163. 10.1080/23311932.2019.1704163

[CR32] Laperche B, Munier F, Hamdouch A (2008) The collective innovation process and the need for dynamic coordination: general presentation. J Innov Econ Manag 2:3–13. 10.3917/jie.002.0003

[CR33] Leakey RRB (1999) Potential for novel food products from agroforestry trees: a review. Food Chem 66:1–14. 10.1016/S0308-8146(98)00072-7

[CR34] Leakey R, van Damme P (2014) The role of tree domestication in green market product value chain development. For Trees Livelihoods 23:116–126. 10.1080/14728028.2014.887371

[CR35] Leakey RRB, Tchoundjeu Z, Schreckenberg K, Shackleton SE, Shackleton CM (2005) Agroforestry tree products (AFTPs): targeting poverty reduction and enhanced livelihoods. Int J Agric Sustain 3:1–23. 10.1080/14735903.2005.9684741

[CR36] Lee BH, Struben J, Bingham CB (2018) Collective action and market formation: an integrative framework. Strat Mgmt J 39:242–266. 10.1002/smj.2694

[CR37] Macqueen DJ (2008) Forest connect: reducing poverty and deforestation through support to community forest enterprises. Int For Rev 10:670–675

[CR38] Macqueen D, Bolin A, Greijmans M, Grouwels S, Humphries S (2020) Innovations towards prosperity emerging in locally controlled forest business models and prospects for scaling up. World Dev 125:104382. 10.1016/j.worlddev.2018.08.004

[CR39] Makate C (2019) Effective scaling of climate smart agriculture innovations in African smallholder agriculture: a review of approaches, policy and institutional strategy needs. Environ Sci Policy 96:37–51. 10.1016/j.envsci.2019.01.014

[CR40] Martin DM, Schouten JW (2013) Consumption-driven market emergence. J Consum Res 40:855–870

[CR41] Meinhold K, Darr D (2019) The processing of non-timber forest products through small and medium enterprises—a review of enabling and constraining factors. Forests 10:1026

[CR42] Menary J, Collier R, Seers K (2019) Innovation in the UK fresh produce sector: identifying systemic problems and the move towards systemic facilitation. Agric Syst 176:102675. 10.1016/j.agsy.2019.102675

[CR43] Mishra AA, Shah R (2009) In union lies strength: collaborative competence in new product development and its performance effects. J Oper Manag 27:324–338. 10.1016/j.jom.2008.10.001

[CR44] Molnar A, Gomes D, Sousa R, Vidal N, Hojer RF, Arguelles LA, Kaatz S, Martin A, Donini G, Scherr S (2008) Community forest enterprise markets in Mexico and Brazil: new opportunities and challenges for legal access to the forest. J Sustain For 27:87–121

[CR45] Momanyi DK, Owino WO, Makokha A, Evang E, Tsige H, Krawinkel M (2019) Gaps in food security, food consumption and malnutrition in households residing along the baobab belt in Kenya. Nutr Food Sci 49(6):1099–1112. 10.1108/NFS-11-2018-0304

[CR46] Mounjouenpou P, Eyenga Ngono, Nina Sophie Natacha, Kamsu EJ, Bongseh Kari P, Ehabe EE, Ndjouenkeu R (2018) Effect of fortification with baobab (*Adansonia digitata* L.) pulp flour on sensorial acceptability and nutrient composition of rice cookies. Sci Afr 1:00002. 10.1016/j.sciaf.2018.e00002

[CR47] Mu J, Thomas E, Peng G, Di Benedetto A (2017) Strategic orientation and new product development performance: the role of networking capability and networking ability. Ind Mark Manag 64:187–201. 10.1016/j.indmarman.2016.09.007

[CR48] Mumbeya PN, Matungula PK, Masuki KK, Schut M, Okafor C (2020) Can innovation platforms (IPs) improve rural women participation in maize value chain? Evidence from the Eastern DR Congo. Eur J Agric Food Sci 2:3

[CR49] Nair PKR, Viswanath S, Lubina PA (2017) Cinderella agroforestry systems. Agrofor Syst 91:901–917. 10.1007/s10457-016-9966-3

[CR50] Nitcheu Ngemakwe PH, Remize F, Thaoge ML, Sivakumar D (2017) Phytochemical and nutritional properties of underutilised fruits in the southern African region. Econ Bot 113:137–149. 10.1016/j.sajb.2017.08.006

[CR51] Omondi M, Rimberia FK, Wainaina CM, Mukundi JBN, Orina J, Gebauer J, Kehlenbeck K (2019) Fruit morphological diversity and productivity of baobab (*Adansonia digitata* L.) in coastal and lower eastern Kenya. For Trees Livelihoods 28:266–280. 10.1080/14728028.2019.1659861

[CR52] Pamuk H, Bulte E, Adekunle AA (2014) Do decentralized innovation systems promote agricultural technology adoption? Experimental evidence from Africa. Food Policy 44:227–236. 10.1016/j.foodpol.2013.09.015

[CR53] Pandit BH, Albano A, Kumar C (2009) Community-based forest enterprises in Nepal: an analysis of their role in increasing income benefits to the poor. Small Scale For 8:447. 10.1007/s11842-009-9094-2

[CR54] Rametsteiner E, Weiss G (2006) Innovation and innovation policy in forestry: linking innovation process with systems models. For Policy Econ 8:691–703. 10.1016/j.forpol.2005.06.009

[CR55] Reed J, van Vianen J, Foli S, Clendenning J, Yang K, MacDonald M, Petrokofsky G, Padoch C, Sunderland T (2017) Trees for life: the ecosystem service contribution of trees to food production and livelihoods in the tropics. For Policy Econ 84:62–71. 10.1016/j.forpol.2017.01.012

[CR56] Rogerson CM (2001) In search of the African miracle: debates on successful small enterprise development in Africa. Habitat Int 25:115–142. 10.1016/S0197-3975(00)00033-3

[CR57] Rudolph MJ (1995) The food product development process. Br Food J 97:3–11. 10.1108/00070709510081408

[CR58] Russell D, Franzel S (2004) Trees of prosperity: agroforestry, markets and the African smallholder. Agrofor Syst 61:345–355. 10.1023/B:AGFO.0000029009.53337.33

[CR59] Sanchez AC, Osborne PE, Haq N (2010) Identifying the global potential for baobab tree cultivation using ecological niche modelling. Agrofor Syst 80:191–201. 10.1007/s10457-010-9282-2

[CR60] Sanou J, Bayala J, Teklehaimanot Z, Bazié P (2012) Effect of shading by baobab (*Adansonia digitata*) and néré (*Parkia biglobosa*) on yields of millet (*Pennisetum glaucum*) and taro (*Colocasia esculenta*) in parkland systems in Burkina Faso, West Africa. Agrofor Syst 85:431–441. 10.1007/s10457-011-9405-4

[CR61] Sanyang S, Taonda SJ-B, Kuiseu J, Coulibaly N’T, Konaté L (2016) A paradigm shift in African agricultural research for development: the role of innovation platforms. Int J Agric Sustain 14:187–213. 10.1080/14735903.2015.1070065

[CR62] Schumann K, Wittig R, Thiombiano A, Becker U, Hahn K (2010) Impact of land-use type and bark- and leaf-harvesting on population structure and fruit production of the baobab tree (*Adansonia digitata* L.) in a semi-arid savanna, West Africa. For Ecol Manag 260:2035–2044. 10.1016/j.foreco.2010.09.009

[CR63] Schut M, Klerkx L, Sartas M, Lamers D, Mmc Campbell, Ogbonna I, Kaushik P, Atta-Krah K, Leeuwis C (2016a) Innovation platforms: experiences with their institutional embedding in agricultural research for development. Ex Agric 52:537–561. 10.1017/S001447971500023X

[CR64] Schut M, van Asten P, Okafor C, Hicintuka C, Mapatano S, Nabahungu NL, Kagabo D, Muchunguzi P, Njukwe E, Dontsop-Nguezet PM, Sartas M, Vanlauwe B (2016b) Sustainable intensification of agricultural systems in the Central African Highlands: the need for institutional innovation. Agric Syst 145:165–176. 10.1016/j.agsy.2016.03.005

[CR65] Seixas CS, Berkes F (2010) Community-based enterprises: the significance of partnerships and institutional linkages. Int J Commons 4:183. 10.18352/ijc.133

[CR66] Shackleton S, Shanley P, Ndoye O (2007) Invisible but viable: recognising local markets for non-timber forest products. Int For Rev 9:697–712. 10.1505/ifor.9.3.697

[CR67] Steiner A, Teasdale S (2019) Unlocking the potential of rural social enterprise. J Rural Stud 70:144–154. 10.1016/j.jrurstud.2017.12.021 31787801 10.1016/j.jrurstud.2017.12.021 PMC6876678

[CR68] Stewart-Knox B, Mitchell P (2003) What separates the winners from the losers in new food product development? Trends Food Sci Technol 14:58–64. 10.1016/S0924-2244(02)00239-X

[CR69] Teklehaimanot Z (2004) Exploiting the potential of indigenous agroforestry trees: parkia biglobosa and Vitellaria paradoxa in sub-Saharan Africa. Agrofor Syst 61:207–220. 10.1023/B:AGFO.0000029000.22293.d1

[CR70] Tenywa MM, Rao KPC, Tukahirwa JB, Buruchara RA, Adekunle AA, Mugabe J, Wanjiku C, Mutabazi S, Fungo B, Kashaija NIM (2011) Agricultural innovation platform as a tool for development oriented research: lessons and challenges in the formation and operationalization. J Agric Environ Stud 2(1):117–146

[CR71] Thomas R, Reed M, Clifton K, Appadurai N, Mills A, Zucca C, Kodsi E, Sircely J, Haddad F, Hagen C, Mapedza E, Woldearegay K, Shalander K, Bellon M, Le Q, Mabikke S, Alexander S, Leu S, Schlingloff S, Lala-Pritchard T, Mares V, Quiroz R (2018) A framework for scaling sustainable land management options. Land Degrad Dev 29:3272–3284. 10.1002/ldr.3080

[CR72] Torri M-C (2010) Community-based enterprises: a promising basis towards an alternative entrepreneurial model for sustainability enhancing livelihoods and promoting socio-economic development in rural India. J Small Bus Entrep 23:237–248. 10.1080/08276331.2010.10593484

[CR73] Tzokas N, Hultink EJ, Hart S (2004) Navigating the new product development process. Ind Mark Manage 33:619–626. 10.1016/j.indmarman.2003.09.004

[CR74] van Wyk B-E (2011) The potential of South African plants in the development of new food and beverage products. Econ Bot 77:857–868. 10.1016/j.sajb.2011.08.003

[CR75] Venter SM, Witkowski ETF (2010) Baobab (*Adansonia digitata* L.) density, size-class distribution and population trends between four land-use types in northern Venda, South Africa. For Ecol Manag 259:294–300. 10.1016/j.foreco.2009.10.016

[CR76] Venter SM, Witkowski ETF (2013) Fruits of our labour: contribution of commercial baobab (*Adansonia digitata* L.) fruit harvesting to the livelihoods of marginalized people in northern Venda, South Africa. Agrofor Syst 87:159–172. 10.1007/s10457-012-9532-6

[CR77] Welford L, Le Breton G (2008) Bridging the gap: phytotrade Africa’s experience of the certification of natural products. For Trees Livelihoods 18:69–79. 10.1080/14728028.2008.9752618

[CR78] Wickens GE, Lowe P (2008) The Baobabs: Pachycauls of Africa, Madagascar and Australia. Springer, Berlin

[CR79] Wynberg R, Laird S, van Niekerk J, Kozanayi W (2015) Formalization of the natural product trade in Southern Africa: unintended consequences and policy blurring in biotrade and bioprospecting. Soc Nat Resour 28:559–574. 10.1080/08941920.2015.1014604

